# Opportunities and challenges for physical rehabilitation with indigenous populations

**DOI:** 10.1097/PR9.0000000000000838

**Published:** 2020-09-23

**Authors:** Ivan Lin, Juli Coffin, Jonathan Bullen, Cheryl Barnabe

**Affiliations:** a Western Australian Centre for Rural Health, University of Western Australia, Geraldton, Australia; b Geraldton Regional Aboriginal Medical Service, Geraldton, Australia; c Telethon Kids Institute, Broome, Australia; d Curtin Medical School, Curtin University, Perth, Australia; e Departments of Medicine and Community Health Sciences, University of Calgary, Calgary, AB, Canada

**Keywords:** Aboriginal, Culture, Appropriate, Disparities, Musculoskeletal, Equity

## Abstract

Indigenous peoples in colonised countries internationally experience a disproportionately high burden of disease and disability. The impact of many of these conditions, such as musculoskeletal pain, can be ameliorated by participating in physical rehabilitation. However, access by Indigenous peoples to physical rehabilitation is low. Overcoming barriers for Indigenous peoples to access high-quality, effective, culturally secure physical rehabilitation should be a priority. Physical rehabilitation outcomes for Indigenous peoples can be enhanced by addressing health system, health service, and individual clinician-level considerations. System-level changes include a greater commitment to cultural security, improving the funding of physical rehabilitation to Indigenous communities, building the Indigenous physical rehabilitation workforce, and developing and using Indigenous-identified indicators in quality improvement. At the health service level, physical rehabilitation should be based within Indigenous health services, Indigenous people should be employed as physical rehabilitation professionals or in allied roles, and cultural training and support provided to the existing physical rehabilitation workforce. For clinicians, a focus on cultural development and the quality of communication is needed. Indigenous ill-health is complex and includes societal and social influences. These recommendations offer practical guidance toward fair, reasonable, and equitable physical rehabilitation outcomes for Indigenous peoples.

## Abstract 2

Indigenous peoples internationally have low access to physical rehabilitation, despite high needs. Achieving equitable outcomes requires reorientating services to be culturally appropriate for Indigenous peoples.

## 1. Background

Equitable access to health services, including physical rehabilitation for all people who need it, is a fundamental human right.^74^ One of the most common reasons to access physical rehabilitation is for persistent pain-related conditions such as low back pain (LBP), osteoarthritis (OA), and persistent pain associated with other musculoskeletal (MSK) conditions. These conditions are common and disabling. Globally, MSK pain is the second largest contributor to disability^19^ and the burden is likely higher than that estimated.^19^ In the 2017 Global Burden of Disease study, LBP was the greatest cause of years lived with disability in 126 of the included 195 countries, including Australia, Canada, and the United States.^48^ Neck pain and other MSK conditions are also leading causes of the disease burden.^48^ Ensuring access to effective, high-quality physical rehabilitation is a cornerstone of management.^57^

Accepting that MSK pain is a significant issue at the population level, it is important to recognize that within individual countries, there are critical disparities in determinants of health, including health service access, for particular population groups. Indigenous peoples originating in lands now called Australia, Canada, the United States, and New Zealand share a common history of colonization, with legacies of ill-health^3^ rooted in assimilation policies^62^ and ongoing structural barriers at several levels of services that impede the attainment of the highest standards of physical and mental health as affirmed in Article 24 of the United Nations Declaration on the Rights of Indigenous Peoples.^73^ There is increasing evidence that conditions that would benefit from physical rehabilitation, in particular MSK pain, have a disproportionately greater impact on Indigenous peoples in these countries.

## 2. Review aims and approach

The aims of this narrative review are to: (1) examine the need for physical rehabilitation amongst Indigenous peoples by summarising the burden of MSK pain conditions and access to physical rehabilitation services, (2) explore the historical and contemporary factors that act as barriers to physical rehabilitation for Indigenous peoples, and (3) outline opportunities to improve physical rehabilitation for Indigenous peoples at system, health service, and clinician levels. Currently, there is a limited body of research in the area of Indigenous MSK pain and physical rehabilitation. For this review, the authors undertook searches of academic and general databases (Medline, CINAHL, PubMed—Aboriginal and Torres Strait Islander health PubMed search tool, and Google Scholar) using terms related to Indigeneity (eg, Indigen* OR Aborigin* Or First [Nations]) and physical rehabilitation (eg, rehabilitat* OR pain). Formal appraisal of studies was not undertaken, although authors were informed by their previous work including undertaking systematic reviews in the field. Authors were also informed by their clinical/professional experiences in Indigenous health care and physical therapy (I.L.), rheumatology (C.B.), psychology (J.B.), and education (J.C.), and for 3 authors, their lived experience as Indigenous people (J.C., J.B., and C.B.). The review focusses on Indigenous peoples in colonised countries with developed economies, such as Australia, Canada, the United States, and New Zealand; however, because of the origins of the author group, we have a particular focus on Australia and Canada. Although the topic of the review is physical rehabilitation, our conclusions may apply to other areas of care that have similar needs, including pain management.

## 3. Burden of pain-related musculoskeletal conditions for indigenous populations

Several systematic reviews of pain conditions amongst Indigenous peoples have included the prevalence and burden of pain conditions summarised from population-level studies. In Australia, the prevalence of LBP amongst Aboriginal Australians is 1.1 times the rate of non-Aboriginal Australians,^58^ and in the United States, American Indian and Alaska Natives reported a higher prevalence of LBP (35 vs 26.4%) and neck pain (20.7 vs 15.3%) when compared to the general or white population.^49^ A systematic review of rheumatic diseases in Indigenous populations of Canada, the United States, Australia, and New Zealand reported that OA affected up to 17% of American Indian/Alaska Native women, 22% of Canadian First Nations people, 32% of Aboriginal Australians, and 6% of New Zealand Maori.^61^ For Aboriginal Australians, this represents a prevalence 1.2 to 1.5 times higher than the general population rate,^58^ and amongst First Nations peoples in Alberta, Canada, the prevalence of OA is nearly double that of the non-First Nation population.^12^ Inflammatory rheumatic diseases also occur at increased rates in Indigenous populations. For Aboriginal Australians, rheumatoid arthritis (RA) occurs at 1.0 to 2.0 times the general population rates,^58^ and for First Nations in Alberta, Canada, the prevalence of RA is 2 to 3 times the non-First Nations populations rates.^13^ Systemic lupus erythematosus prevalence was also disproportionately high.^14^ A review of pain amongst American Indian, Alaska Native, and Aboriginal Canadian peoples reported a higher prevalence of pain and painful conditions than non-Indigenous North Americans.^49^ Pain is also more prevalent amongst Indigenous children and adolescents in the United States when compared to non-Indigenous people of similar age.^53^

The burden of MSK pain related to function and disease experience is accentuated for Indigenous peoples when compared to non-Indigenous peoples. In Australia, the overall burden of MSK pain conditions amongst Indigenous Australians, expressed by Disability Adjusted Life Years, was 1.4 times higher compared to non-Indigenous Australians,^9^ although the burden attributable to OA was equal, and was 30% lower for LBP. The impact of RA on Indigenous North American peoples is reported to be greater in several domains including patient-reported pain, function, fatigue, quality of life, and well-being.^46^

Although the overall burden is higher, this is not associated with increased access to health care. In the general population of Australia, 17.4 per 100 of all primary care encounters are for MSK pain, whereas it is significantly less at 13.6 encounters per 100 amongst Indigenous Australians.^21^ Specific to OA, use of primary care is about half the rate for Indigenous Australians with 3.2 vs 6.5 per 1,000 primary care encounters for knee OA and 1.2 vs 2.3 per 1,000 encounters for hip OA.^58^ By contrast, First Nations people in Alberta^12^ and Manitoba^11^ access primary care at nearly twice the rate as non-Indigenous Canadians for OA. Access to specialised medical care for OA is lower, however, with Aboriginal Australians and First Nation Albertans undergoing total hip or knee replacement surgery at less than half the rate of non-Aboriginal populations.^12,58^ No known studies have reported access to physical rehabilitation by Indigenous peoples, although access to physiotherapists, who are primary providers of physical rehabilitation, is limited. In Australia, physiotherapy services are only available at 52% of Aboriginal and Torres Strait Islander primary healthcare services.^7^ Access to physiotherapists by Indigenous Canadians is also limited.^24^ Collectively, accumulating evidence points to disparities in the epidemiology, impact, and access to care by Indigenous peoples for health conditions that would benefit from physical rehabilitation.

## 4. Barriers to physical rehabilitation for indigenous people

Barriers for Indigenous peoples to access health care are intertwined with the context of colonisation and its sequelae, including the marginalisation and disadvantage experienced by Indigenous peoples, and societal and institutionalised racism. These factors play out in the way healthcare services are delivered and result in a range of cultural, geographical, financial, and health service delivery barriers experienced by Indigenous patients, and ineffective relationships between Indigenous patients and health clinicians.

The negative impact of colonisation on the health of Indigenous peoples has been well documented,^31,60,68^ and a comprehensive review is beyond the scope of this article. However, the history of conflict between Indigenous peoples and colonisers and the many years of sanctioned efforts to diminish Indigenous culture and identity through the forced removal of children, banning the use of traditional languages, and separation of families from country and ancestral homelands reverberates on physical and mental well-being today.^51^ In Australia, the “Bringing them Home” report concluded that in the period of 1910 to 1970, “not one Indigenous family has escaped the effects of forcible removal.”^45^ In Canada, the Royal Commission on Aboriginal Peoples^34^ and the Truth and Reconciliation Commission of Canada reports^64^ summarize the decimation of the Indigenous population and attempts to assimilate the population. The consequences on health and well-being are intergenerational and persistent; the children and grandchildren of those who were forcibly removed are at greater risk of poorer mental and social outcomes.^17^ For these reasons, colonisation has been described as the “primary distal determinant of Indigenous ill-health.”^60^

The historical context sets the scene for a high burden of illness and why Indigenous peoples may distrust mainstream institutions such as healthcare services. Distrust is one of many reasons why Indigenous peoples choose not to engage in mainstream health care. This distrust is fostered by continuing experiences of racism.^2^ In a qualitative study of First Nation Albertans with arthritis, Thurston et al.^71^ reported that a primary reason participants did not access specialist care was “prior negative experiences with racism in the healthcare system.” In an Australian nationally representative sample, 53% of Indigenous Australians reported experiencing racism when seeking health care, the highest of any identified group.^18^ Indigenous Australians also reported the highest experiences of everyday racism including being treated less respectfully, being perceived that you are not to be trusted, or insulted.^18^ Although these reflect examples of overt interpersonal racism, institutional racism in health care may be multilevel, pervasive, and go unrecognised by healthcare services or providers. At a health system level, this includes a lack of Indigenous people in healthcare leadership, health care that privileges Western perspectives of health and deprioritises Indigenous healing approaches, and a lack of Indigenous health staff.^38,60^ At a health service level, institutionalised racism could include non-Indigenous staff who are not culturally aware and healthcare environments that do not accommodate the needs of Indigenous patients.^67^ The consequences are health services that do not feel culturally secure, nor accessible, for Indigenous peoples.

Adverse experiences of health care are reflected in the experiences of Indigenous peoples who have sought care for conditions requiring physical rehabilitation. Healthcare practitioners and Indigenous patients may have differing viewpoints of the concept of health or health care. Jimenez et al.^49^ reported how American Indian women in the Pacific Northwest conceptualised pain in relation to overall well-being that encompassed mind, body, emotion, and spirit, in contrast to health providers who viewed pain as a physical phenomenon that existed in isolation from other aspects of life.^49^ Ironically, Indigenous world views about pain and health that emphasise the interconnectedness of body, mind, spirit, community, and country align strongly with contemporary biopsychosocial models of pain.^47^ Indigenous Albertans reported “toughing out” pain associated with arthritis due to the demands of managing multiple chronic diseases, social roles, as well as a history of unhelpful healthcare experiences.^71^ By contrast, health practitioners attributed low levels of access to specialist care because Indigenous patients did not “buy-in.” In one qualitative study, Aboriginal Australians in Queensland were reported to “put on a brave face” when living with pain and choose not to seek care.^70^ Again, a reluctance to report pain was also reinforced by poor prior experiences of health care.^70^

Two systematic reviews from Australia^58^ and North America^49^ identified ineffective communication between Indigenous patients and health providers as a primary barrier in pain/MSK pain care because it diminishes the quality of care, does not foster effective relationships between clinicians and patients, and deters Indigenous people from accessing healthcare services. Ineffective communication includes communication that is not congruent with Indigenous peoples' experiences, the use of medical jargon by practitioners, and an absence of communication.^58^ In Australia, cultural and language barriers between patients and practitioners are greatest in remote areas where, despite these issues, there has been limited utilisation of interpreter services.^25^ Other communication barriers can include differences in how pain descriptors such as “ache” or “discomfort” are interpreted by patients and practitioners.^49^ A study amongst Ojibwe women in Manitoba, Canada, also reported that participants may be unwilling to discuss pain as a way of coping with it.^49^

Geographical issues may be a barrier to accessing physical rehabilitation. Although a greater number of Indigenous people live in urban than nonurban areas, Indigenous peoples make up a great proportion of the population the further one moves away from urban areas. In Australia, for example, 35% of Indigenous Australians live in urban areas and 14% in very remote areas, compared to 71% and 0.5%, respectively, of the non-Indigenous population.^8^ This is accompanied by a gradient of healthcare access with reduced health care the further one moves away from major cities. In Australian major capital cities, there are 107.8 physiotherapists per 100,000 people, compared to 50.9 and 44.9 physiotherapists in remote or very remote areas, respectively.^6^ With increased remoteness, there are fewer physiotherapists per capita who provide health care over a greater geographical area to populations with poorer overall health and a higher burden of illness. Similar geographical barriers exist in Canada.^24^ These factors result in a challenging context in which to provide physical rehabilitation.

Currently, little is known about the quality of physical rehabilitation provided to Indigenous peoples; however, disparities in the management and outcomes of pain conditions between Indigenous and non-Indigenous patients have been reported. In Australian primary care, Aboriginal patients are twice as likely to be prescribed an opioid for pain as compared to non-Aboriginal Australians.^44^ In the Canadian Early Arthritis Cohort, Indigenous Canadians diagnosed with RA experience poorer outcomes than non-Indigenous patients.^63^ The authors suggest that RA outcomes could be improved by offering culturally appropriate care that addresses the complexity of RA presentations, including social determinants of health.

## 5. Opportunities to improve physical rehabilitation access and outcomes for indigenous peoples

A comprehensive approach to improving physical rehabilitation outcomes for Indigenous peoples is multilevel and includes addressing upstream determinants of health associated with social disadvantage and discrimination. Addressing the social determinants of health is beyond the scope of this article and, in general, physical rehabilitation services. However, understanding the sociocultural context of the population who require physical rehabilitation, or indeed each individual patient's situation, is key to delivering high-quality, patient-centred care.

Our focus below is to consider how physical rehabilitation outcomes for Indigenous peoples can be enhanced. We address considerations at system, health service, and clinician levels.

### 5.1. System-level considerations

System-level considerations relate to the broader environment in which health care, including physical rehabilitation services, exist. This includes policy and regulation, financial/payment factors, workforce planning, information systems, and mechanisms to optimise service delivery.^20^ System considerations include an overarching commitment to cultural security, availability of funding to support physical rehabilitation, increasing the number of Indigenous physical rehabilitation workers, Indigenous health curricula for physical rehabilitation professionals, and identifying and using indicators to monitor and Indigenous physical rehabilitation care.

At a system level, cultural security is supported by policies that formalise how physical rehabilitation can be readily accessed by Indigenous clients, and cultural practices are in-built into services.^26^ At a minimum, policy related to cultural security should recognise the ongoing impact of colonisation, and ensure there is multilevel Indigenous representation within health care, from leadership to health care delivery levels,^40,66^ and mechanisms by which patient input continuously serves to improve service environments. Evaluation of outcomes and identification of interventions to resolve inequities are paramount in closing care gaps. This commitment should be reflected through all levels of the health system, from administration down through to frontline service provision, and will require redistribution of power and resources from those holding privilege.

The availability of funding to support physical rehabilitation is an important consideration. We will not address funding models in detail; however, we appreciate that access to physical rehabilitation may be constrained by systems that prioritise acute or primary care over physical rehabilitation. Furthermore, even within the same country, Indigenous peoples may have different levels of access to health care, or there may be variations in funding programs between states/territories, health regions, or jurisdictions.^24^ Understanding the opportunities or constraints associated with funding may influence the planning and delivery of physical rehabilitation services at a community or individual patient level. There are a number of reasons why ensuring Indigenous health services are resourced to provide culturally secure physical rehabilitation to optimise outcomes. These are discussed further below.

The physical rehabilitation workforce should be matched to needs in Indigenous communities. This involves increasing the number of Indigenous physical rehabilitation professionals, and cultural curricula for those entering the physical rehabilitation professions, either as students or for those trained elsewhere. Currently Indigenous people are underrepresented, or non-Indigenous people are overrepresented, in the physical rehabilitation workforce. For example, in 2012, only 0.4% of Australia's physiotherapy workforce identified as Aboriginal or Torres Strait Islander.^42^ Indigenous peoples are also underrepresented in Canadian physiotherapy education programs.^27^ In a review of recruitment of Indigenous students into tertiary health education, Curtis et al. recommended a comprehensive “pipeline” supporting Indigenous student at preentry, transitioning to tertiary study, completing tertiary qualification, and in postgraduate development, underpinned by institutional commitment to equity.^30^ Engaging communities and families, particularly at the preentry stage, and research and evaluation to examine outcomes were also recommended.^30^ All those entering the physical rehabilitation professions, as students or through license examinations if trained elsewhere, should demonstrate base-level competence to work effectively in Indigenous health settings. This should be supported by overarching Indigenous health curricula^41^ that, in addition to Indigenous health and cultures, includes a critical examination of the health professions, the culture and expectations of that discipline, and how this relates to delivering physical rehabilitation with Indigenous peoples, unrecognised “cultural artefacts” within health disciplines such as the “hidden” curriculum and its influence on student and professional capabilities,^65^ and the shaping of professional identity upon and beyond graduation.^29^

Indicators are measures of the structures, processes, or outcomes of care so that healthcare performance can be monitored and improved.^59^ Indicators are used to increase the quality of Indigenous health care through continuous quality improvement, involving cycles of clinical audit of indicators, analysis, collaborative interpretation and goal setting, and implementing actions.^10^ This process needs to be supported by suitable information technologies.^40^ At present, these measures and indicators are typically Western in nature, focus, and implementation, and predicated on a transactional model of ways of knowing and doing.^22^ A simple indicator is to measure the access and uptake of Indigenous people to physical rehabilitation services. Additional physical rehabilitation indicators should be identified by Indigenous peoples, with a commitment of health systems to record and collaboratively plan, with Indigenous people, both strategies for improvement and the measures of improvement themselves. This collaborative process, with a focus on partnerships and Indigenous peoples' needs, is potentially transformative on the systems that capture and store these data and involved stakeholders.

### 5.2. Health service-level considerations

To optimise outcomes, physical rehabilitation services should be based within Indigenous health services, employ Indigenous people as physical rehabilitation professionals or in allied roles, and support the existing physical rehabilitation workforce with cultural training.

Culturally secure health care is most commonly delivered through Indigenous healthcare services. Indigenous primary health care services have specialised expertise in Indigenous health, offer comprehensive health care, are Indigenous community controlled, and provide employment and training for Indigenous people, and understand the social and cultural determinants of Indigenous health.^23,33^ Physical rehabilitation should be offered in Indigenous health services because they maximise access to care, are more familiar to Indigenous peoples, and trusted by Indigenous peoples. A new rheumatology service introduced within the Elbow River Healing Lodge, Calgary, Canada, increased access to specialist rheumatology care by reducing barriers for patients.^15^ Indigenous patients reported the service to be highly acceptable and culturally safe. Offering cardiac rehabilitation and psychiatric care within Indigenous health services has also been reported to increase accessibility and acceptability to Indigenous patients.^35,54^ If Indigenous health services do not have the resources to provide “in-house” physical rehabilitation, then partnerships between mainstream physical rehabilitation services and Indigenous health services are recommended.

Currently, there are too few Indigenous people working in physical rehabilitation either as physical rehabilitation professionals or in complementary roles. In addition to delivering clinical care, roles for Indigenous people in health settings includes cultural and community brokerage, educating non-Indigenous healthcare staff, and language interpretation. Employing Indigenous people in health care improves Indigenous health outcomes.^1^ Increasing the Indigenous physical rehabilitation workforce can increase access to care and the quality of care by reducing cultural barriers and making health care more welcoming for Indigenous peoples.^5^ A recent systematic review examined factors that assisted retention of Indigenous Australians within the health workforce.^52^ These factors can inform planning for an Indigenous physical rehabilitation workforce and include a supportive, respectful, and flexible work environment; access to clinical and cultural mentorship; clearly defined roles, scope of practice, and responsibilities; and appropriate remuneration.^52^

Currently, the physical rehabilitation workforce is predominantly non-Indigenous, and cultural education and development is needed to enhance the cultural security of care. A systematic review of systematic reviews of interventions to improve cultural competence reported moderate evidence that cultural training improves healthcare provider outcomes and increases care utilisation, and weaker evidence of improved patient outcomes.^72^ Cultural training should build upon cultural curricula described above, be tailored to the skills needed for specific professional groups, and ideally accompanied by systemic and organisational commitment to change. Cultural awareness training, in which participants learn about “other” cultures, has been criticised because of the potential to increase stereotype beliefs and reinforce essentialist racial identities.^72^ Instead a critical, self-reflective approach is recommended that acknowledges institutional racism^38,43^ is ongoing as opposed to one-off training sessions, and includes a practical approach of how cultural security is demonstrated in practice.^37^

### 5.3. Clinician-level considerations

Although implementing sustainable high-quality physical rehabilitation to Indigenous patients involves changes at a system and health service level discussed above, this should not undervalue the important role clinicians' play in individual patient experiences of physical rehabilitation. Developing strong and trusting relationships with Indigenous patients is one of the most powerful ways physical rehabilitation professionals can encourage engagement by Indigenous patients over time.^32^ Key considerations are taking time to develop relationships with patients and the community, cultural development, and enhancing the effectiveness of communication.

Patient-centred care encompasses the whole person, whereby people are considered within their individual context, and their knowledge, needs, values, goals, and preferences are understood and codeveloped into shared decision-making about management.^39^ Davy et al.^32^ argue that optimal care for Indigenous people with chronic disease extends beyond patient-centred care and also includes taking time to build relationships with the community and understanding the social and cultural lives of patients. The process of developing relationships takes time to develop trust and effective communication. Conversely, the experiences of racism and stereotyping erodes trust; the experience of Indigenous peoples being judged based on race-based stereotypes has been reported extensively in health care and impacts negatively on healthcare experiences.^4,5,32^ For physical rehabilitation clinicians who work with Indigenous patients, understanding patients and the community is part of a cultural development process.

Personal cultural development is a way for clinicians to work toward delivering high-quality and culturally secure physical rehabilitation. For clinicians, and as has been discussed above, we propose that a commitment to learn about culture, one's own and others', and how culture impacts on health and health care, is a useful starting point.^36^ This involves being culturally curious, reflective, and seeking a range of cultural learning opportunities. Examples include formal education programs, eg, cultural awareness or antiracism training; immersive experiences, eg, visiting an Indigenous community or organisation; seeking informal experiences, eg, talking with Indigenous work colleagues or patients; or other cultural participation, eg, celebrating cultural festivals or contributing to reconciliation activities within the workplace. To guide cultural development, a comprehensive framework that incorporates knowledge/skills/experience, attitude/values, and actions may be helpful.^28^

A key skill for high-quality physical rehabilitation, or any health care, is effective communication, and is especially important in Indigenous health care.^5,38,59^ Effective communication is critical in health care and to develop successful relationships between patients and clinicians.^69^ The effectiveness of communication is a primary determinant of why Indigenous people with MSK pain choose to engage or disengage with health care.^58^ Communication barriers for Indigenous people exist across all areas of health care including chronic disease,^5^ cardiac disease,^4^ kidney disease,^25^ end-of-life care,^50^ and cancer.^67^ Clinicians need to recognise when communication is not effective, and develop skills and capabilities to improve upon this.

Patient-centred communication frameworks for Indigenous health care may be useful to clinicians. In Australia, one framework is “clinical yarning,” which reconceptualises clinical communication as a yarn.^55^ Yarning is a term used by Aboriginal Australians and refers to a relaxed style of communication that is two-way, in which power is shared, and in which each party is accountable to the other.^16^ Clinical yarning reframes clinical communication into 3 areas: the social yarn, which is a way to develop the relationship between patient and practitioner; the diagnostic yarn, in which clinical information is gathered in a conversational, narrative style; and the management yarn, in which health issues are explained in ways that are personally meaningful and used as a platform to collaboratively develop a management plan.^55^ Clinical yarning approaches may require more time initially with time requirements reducing once a relationship has developed. Alternatively, it is likely that culturally informed communication approaches such as clinical yarning, like other patient-centred approaches,^69^ are more time-efficient because they get to a patient's main concern more quickly. To improve communication, active learning strategies such as role play, learner interaction, practice, and feedback are needed.^56^

## 6. Conclusion

Reducing the high burden of disability and disease amongst colonised Indigenous communities internationally is a complex issue. Despite the high burden, Indigenous peoples face barriers to accessing physical rehabilitation; enhancing access to high-quality physical rehabilitation should be a priority. To enhance physical rehabilitation outcomes with Indigenous peoples, changes at system, health service, and individual clinician levels should be considered. System-level changes include a system-wide commitment to cultural security, examining funding availability of physical rehabilitation, building the Indigenous physical rehabilitation workforce, and developing and using Indigenous-identified indicators in quality improvement. At the health service level, physical rehabilitation should be based within Indigenous health services, Indigenous people should be employed as physical rehabilitation professionals or in allied roles, and cultural training and support provided to the existing physical rehabilitation workforce. For clinicians, building relationships with patients and communities, a focus on cultural development and the quality of communication is needed. A focus on improving physical rehabilitation to Indigenous peoples is needed if fair, reasonable, and equitable outcomes are to be achieved.

## Disclosures

The authors declare there are no conflicts of interest.
